# SPECT Imaging of Acute Disc Herniation by Targeting Integrin α5β1 in Rat Models

**DOI:** 10.3389/fneur.2022.782967

**Published:** 2022-05-09

**Authors:** Jian Guan, Chenghua Yuan, Xin Tian, Lei Cheng, Hannan Gao, Qingyu Yao, Xinyu Wang, Hao Wu, Zan Chen, Fengzeng Jian

**Affiliations:** ^1^Department of Neurosurgery, Xuanwu Hospital, Capital Medical University, Beijing, China; ^2^Spine Center, China International Neuroscience Institute (CHINA-INI), Beijing, China; ^3^Research Center of Spine and Spinal Cord, Beijing Institute of Brain Disorders, Capital Medical University, Beijing, China; ^4^National Center for Neurological Disorders, Xuanwu Hospital, Beijing, China; ^5^Center for Experimental Animals, Xuanwu Hospital, Capital Medical University, Beijing, China; ^6^Medical Isotopes Research Center and Department of Radiation Medicine, State Key Laboratory of Natural and Biomimetic Drugs, School of Basic Medical Sciences, Peking University, Beijing, China

**Keywords:** molecular imaging, integrin α5β1, ^99*m*^Tc-3PisoDGR2, disc herniation, acute

## Abstract

**Objective:**

Traditional morphological imaging of intervertebral disc herniation (IVDH) is challenging in early disease diagnosis. Aiming at the early diagnosis of IVD by non-invasive molecular imaging targeting of integrin α5β1, we performed novel imaging in rats with acute IVDH for the first time.

**Methods:**

Animal models were prepared by conducting an established needle puncture procedure through the normal intervertebral disc (IVD). The disc-injured rats underwent SPECT/CT imaging of the ^99m^Tc-3PisoDGR2 peptide at 1 day to 2 months postinjury. The expression change of integrin α5β1 was determined by anti-integrin α5 and anti-integrin α5β1 immunohistochemistry (IHC). Magnetic resonance imaging (MRI) was performed for comparison during disease progression. The morphological changes of the disc were determined by safranin-O staining.

**Results:**

Rats with acute IVDH showed gradually increased disc uptake of ^99m^Tc-3PisoDGR2 from 1 to 7 days posttreatment, which was a significantly higher level than that of the normal disks in degenerative diseases. IHC results showed the expression of integrin α5β1 on the surface of annulus fibrosus (AF) cells and nucleus pulposus (NP) cells, which agreed with the uptake data. MRI showed a progressively decreased T2 density and MRI index throughout the investigation. Hematoxylin and eosin (HE) staining and safranin-O staining revealed a disorganized structure of the IVD as well as loss of proteoglycans after puncture.

**Conclusions:**

The present study demonstrated a good correlation between integrin α5β1 expression and acute disc herniation. The SPECT/CT imaging of ^99m^Tc-3PisoDGR2 targeting integrin α5β1 may diagnose IVDH in an acute phase for early disease management.

## Introduction

Lumbar disc herniation, a common disease that causes low back pain (LBP) (1), is a worldwide issue leading to human incapacity (2). To avoid irreversible degenerative disc herniation, early diagnosis of intervertebral disc (IVD) pathological changes is crucial for disease management in quinquagenarians who are at high risk (3, 4). Although traditional computed tomography (CT) and magnetic resonance imaging (MRI) are the most common noninvasive strategies for the diagnosis of lumbar disc diseases (5), advice based on morphological changes at the early stages of disc herniation remains elusive (6). Compared to anatomical imaging, molecular imaging provides precise and specific information for detecting disease in the early stage.

Strategies for the molecular diagnosis of disc herniation by certain biomarkers are of interest (7). Unlike most normal tissues with high contents of blood vessels and nerves, the nucleus pulposus (NP) in the IVD possesses a high content of matrix cells and extracellular matrix (8, 9). Fibronectin, a classical extracellular matrix component, is highly expressed by local disc cells during disc degeneration (10), and its receptor, integrin α5β1, plays an important role in regulating inflammatory cytokines produced by human articular chondrocytes (11). Therefore, integrin α5β1 may be a biomarker for the early diagnosis of disc herniation. However, traditional immunohistochemistry (IHC) tests by invasive biopsies or surgery are not practical in spines, but molecular imaging can assess receptor expression in a real-time and non-invasive manner (12, 13). Moreover, peptide imaging probes show rapid clearance from circulation, good tissue penetration, and high specificity for real-time imaging (14).

Recently, molecular imaging targeting of integrin α5β1 by single-photon emission computed tomography (SPECT) or positron emission tomography (PET) imaging has been investigated for tumor and rheumatoid arthritis diagnosis (15–17). However, molecular imaging targeting of integrin α5β1 in disc herniation has not been reported. Recently, we reported a radiolabelled isoDGR dimer peptide of ^99m^Tc-3PisoDGR_2_ for SPECT/CT imaging to diagnose integrin α5β1-positive tumors (18). In the present study, we hypothesized that high integrin α5β1 expression in intervertebral disks indicates pathological changes at the molecular level for detecting early lumbar disc herniation. Therefore, we applied SPECT/CT imaging of ^99m^Tc-3PisoDGR_2_ in acute disc herniation rat models. In contrast to traditional MRI, this imaging strategy showed evident advantages in the early diagnosis of disc herniation.

## Materials and Methods

### Animals

A total of 26 male Sprague-Dawley rats (12 weeks old; 250–300 g) were purchased from the Experimental Animal Research Center, Chinese Academy of Medical Science, Beijing, China. The rats were housed in standard sterile pathogen-free facilities (temperature, 20 ± 2°C; humidity, 50–60%; and 12-h light/dark cycle) and given free access to food and water. In the experimental group (14 rats in total), five rats were evaluated by imaging, and samples from the other nine rats were collected for histology at different time points after the operation. In the control group (12 rats in total), three rats were evaluated by imaging, and samples from the other nine rats were collected for histology at different time points after the operation. The present study was approved by the Institutional Review Board (XWH20200206), and the animal care complied with the Guide for the Care and Use of Laboratory Animals.

### Study Design

**Figure 1:**
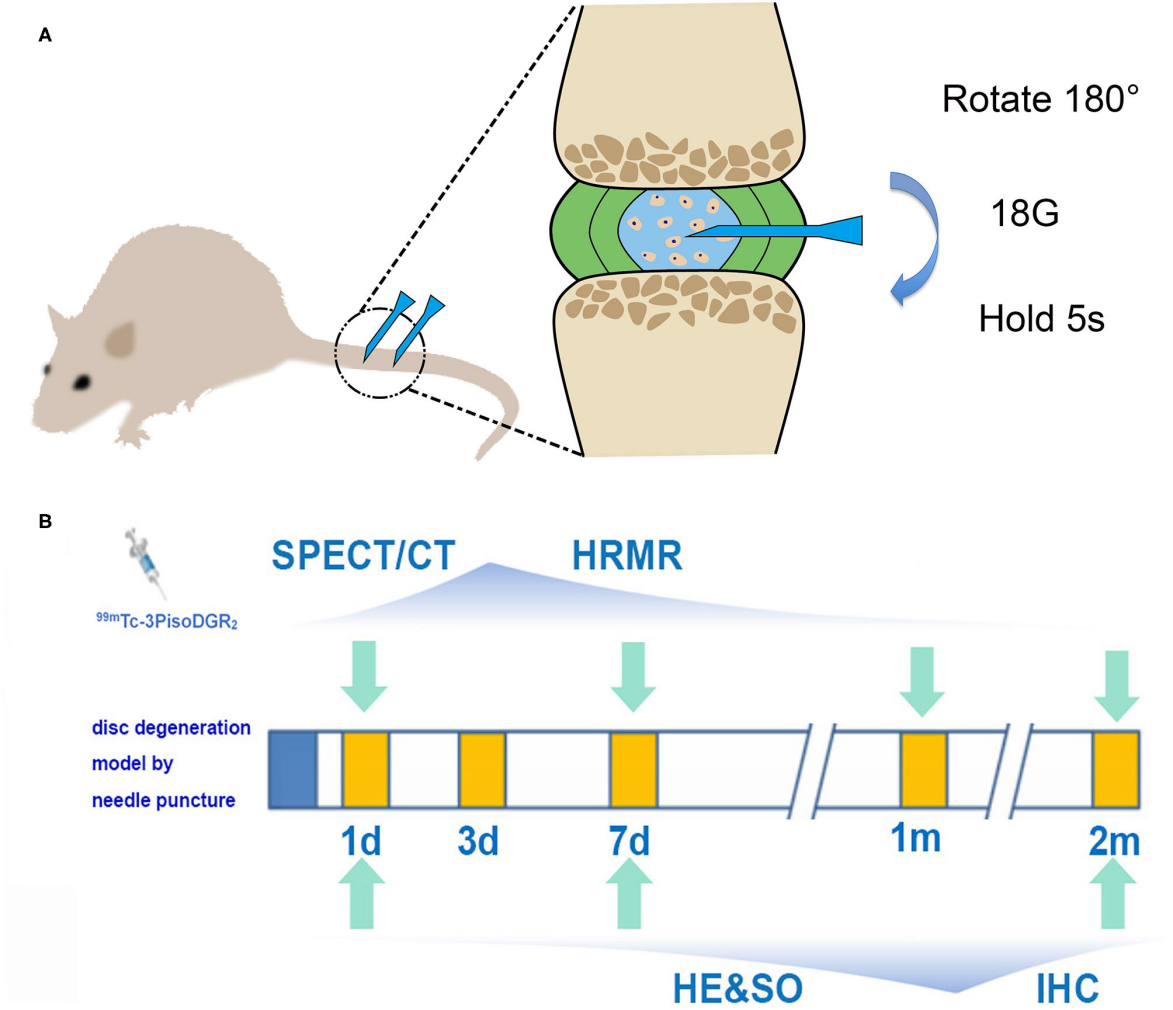
**(A)** Schematic diagram of the model of disc herniation induced in rats by conducting an established needle puncture procedure. **(B)** Experimental flowchart. SPECT and MRI were performed 1 day, 1 week, 1 month, and 2 months postinjury. Histology was performed 1 day, 1 week, and 2 months postinjury (IHC, immunohistochemistry; HRMR, high-resolution MRI; HE, hematoxylin and eosin; SO, safranin-O staining).

The experimental flowchart is presented in Figure 1. The grouping information and number of rats at each checkpoint are listed in Table 1.

**Table 1:** Grouping information (*N* = 26 in total).

	**No. of rats at each checkpoint**								
	**Post-op 1-day**		**Post-op 1-week**		**Post-op 1-month**		**Post-op 2-month**		**In total**
	**Group 1 injury**	**Group 2** **sham**	**Group 1 injury**	**Group 2** **sham**	**Group 1 injury**	**Group 2** **sham**	**Group 1 injury**	**Group 2** **sham**	
MRI and SPECT^a^	5	3	5	3	5	3	5	3	8
Histology^b^	3	3	3	3	N/A	N/A	3	3	18

a *Five rats and three rats were assigned to injury group and sham group, respectively. SPECT/CT imaging was performed one day after each MRI time point*.

b *Three rats from each group were sacrificed at each checkpoint*.

*MRI, Magnetic Resonance Imaging; SPECT, Single Photon Emission Computed Tomography*.

### Surgical Procedure

Rats were anesthetized by inhaling isoflurane gas (2% enflurane in 70% nitrous oxide and 30% oxygen), and the surgery was performed according to the routine procedures (19). Briefly, after palpation to locate the caudal levels, a needle (18-gauge with an outer diameter of 0.84 mm) was inserted into the middle part of the NP at Co5/6 and Co6/7 while being guided by C-arm X-ray (GE Brivo OEC850, GE Company, Boston, USA). The needle was rotated 180° and held for 5 s. The depth of penetration was controlled by locking forceps (clamped 5 mm from the tip of the needle). We just put the needle into the skin, and the NP of Co4/5 and Co7/8 remained undisturbed as the control reference.

### Magnetic Resonance Imaging Measurements

Magnetic resonance imaging was performed 1 day, 1 week, 1 month, and 2 months postoperatively (7.0-T MRI scanner, Bruker, Germany, interfaced with Siemens software). Briefly, after inhaling anesthesia, the animal was laid prone, and the tail was straightened in a double-tuned volume radiofrequency coil. Eight serial T2-weighted sagittal images covering the entire disc area were acquired using a spin-echo sequence. The T2WI sequence parameters were as follows: fat saturation, on; repetition time/effective echo time, 3,000/30 ms; field of view, 30 mm × 60 mm; matrix, 128 × 128; slice thickness, 0.5 mm; slice spacing, 0 mm; number of slices, 8; and number of scans, 1 (total scan time = 6.24 min) (7).

Quantitative analysis of the obtained image slices was performed using Analyze 7.0 (AnalyzeDirect). The nucleus region was segmented from the sliced images. Image reconstruction and volume rendering procedures were performed to generate volumetric images and to calculate each nuclear volume. T2-weighted density and MRI index (the area of NP × average signal intensity) were quantified and calculated (7). All image assessments were conducted by three observers independently who were blinded to the study grouping.

### SPECT/CT Imaging

In this study, we used two SPECT probes that target different integrins. The integrin α5β1-targeted ^99m^Tc-3PisoDGR2 was prepared according to our previous studies (15, 18, 20). To prepare ^99m^Tc-3PisoDGR2, a mixture of 20 μg of HYNIC-3PisoDGR2, 6 mg of TPPTS (J&K Chemical Ltd., Beijing, China), 5 mg of tricine, and 74 MBq Na^99m^TcO4 was reacted in 50 mM succinate buffer (pH = 5.0) at 99°C for 15 min. The radiochemical purity (RCP) was determined by radioactive reverse-phase high-performance liquid chromatography (RP-HPLC), resulting in 95% RCP before *in vivo* applications. The integrin αvβ3-targeted ^99m^Tc-3PRGD2 peptide was prepared according to the literature (21), resulting in 95% RCP before *in vivo* applications.

To explore the integrin expression level changes during the progress of acute disc herniation disease, rats were undergoing ^99m^Tc-3PisoDGR2 or ^99m^Tc-3PRGD2 SPECT/CT imaging at 1 day, 1 week, 1 month, and 2 months. Rats were anesthetized with 2% isoflurane, a surgical incision was made in the inner thigh, and vena femoralis injection for 74 MBq ^99m^Tc-3PisoDGR2 or ^99m^Tc-3PRGD2, suturing was performed after injection. SPECT/CT imaging was using a Nano SPECT/CT imaging system (Mediso Ltd., Hungary). SPECT/CT imaging was performed at 0.5-h post-injection. SPECT was performed using four parallel aperture collimators (peak: 140 keV, 20% width; frame time of 20 s), and CT was performed using a helical scan (50 kVp; exposure time of 300 ms). The SPECT and CT images were merged using Nucline software v2.0 (Mediso Ltd.). The SPECT/CT data were analyzed using ASIPro software (version 3.2). The SPECT quantification was determined by ratios of probe uptake (T/NT) of the injured IVD to the adjacent normal intervertebral disc.

### Histology

The number of sacrificed rats for each time point is shown in Table 1. Coccygeal spinal columns were dissected, postfixed in 10% paraformaldehyde overnight, and decalcified with 0.25 M ethylenediaminetetraacetic acid (EDTA) for 3 days. The specimens were embedded in paraffin wax blocks. Median sagittal 5-μm sections were cut with a microtome and mounted on glass slides. The sections were air-dried for at least 4 h before staining. Sections were stained with hematoxylin and eosin (HE) and 0.1% Safranin-O to confirm the degenerative changes in the IVD after needle puncture, including the changes in morphological structure and proteoglycan expression.

### Immunohistochemistry

Immunohistochemistry was performed to determine the presence of protein and the localization of integrins α5 and β1. Sections were incubated with diluted rabbit polyclonal anti-integrin α5 antibody (1:100; Santa Cruz Biotechnology) or diluted rabbit monoclonal anti-integrin β1 antibody (1:100; Epitomics) at 4°C overnight. The signal was visualized with 3,3′-diaminobenzidine (DAB). The sections were imaged using an image analysis system (Leica TCS SP2, Leica, Wetzlar, Germany). Immunoreactive cells were quantified by calculating the average value of three slides (8 μm thick and 80 μm interval) using ImagePro Plus software. The number of α5-positive and β1-positive cells in the studied regions was counted with a × 40 objective. Three distinct field-of-views were randomly selected for counting from anterior edges adjacent to the punctured annulus.

### Statistical Analysis

All data are presented as the mean ± standard deviation (SD). SPSS 22.0 (IBM SPSS, Armonk, NY, USA) was used for statistical analysis. Radioactivity of the disc injury region *in vivo* and MRI data were analyzed by repeated-measures ANOVA with Tukey's multiple comparisons test. Statistical analysis for IHC was performed using one-way ANOVA with Tukey's multiple comparisons test. The *p* < 0.05 was considered statistically significant. Origin 8.5 (Northampton, MA, USA) was used to generate the graphs.

## Results

### *In vivo* Imaging of Integrin α5β1 Expression by Non-invasive SPECT

**Figure 2:**
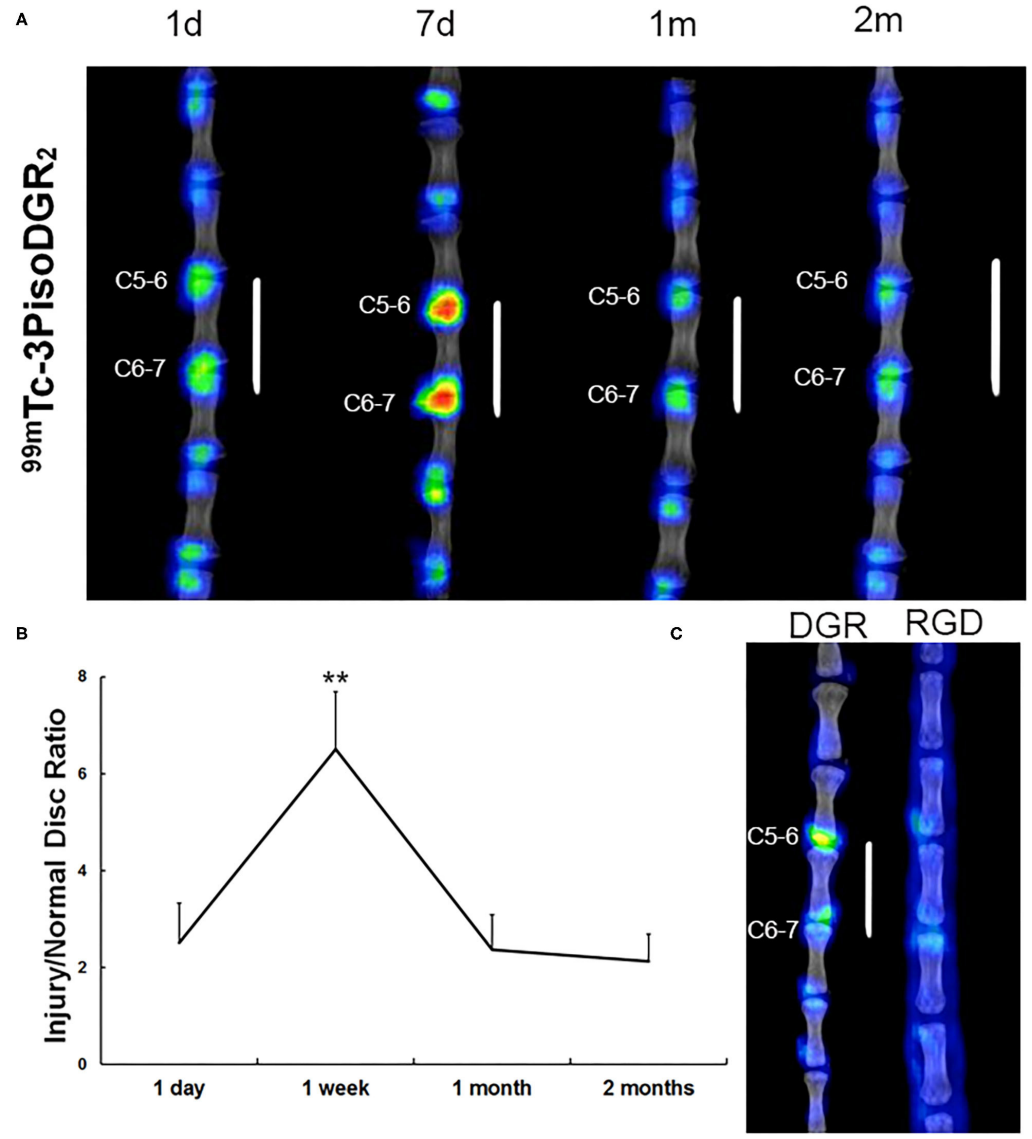
Representative SPECT images in the caudal spine at 1 day, 7 days, 1 month, and 2 months postinjury. **(A)** Radioactive signal accumulation was revealed in the punctured disc area of the injured disc at 1 day postinjury. The radioactivity peaked at 1 week and remained at a higher level until 2 months postinjury. A small amount of radioactivity was found in normal disks. **(B)** The radioactivity of the disc injury region *in vivo* was plotted against the post probe injection time. **p < 0.01 compared to injured disks at 1 day, 1 month, and 2 months. **(C)** To validate the specificity of ^99m^Tc-3PisoDGR2 for integrin α5β1, we conducted ^99m^Tc-RGD imaging in a rat model of disc herniation. ^99m^Tc-RGD is the specific probe for integrin αvβ3, which was intravenously injected into the injury group (*n* = 2) on day 7 postinjury. The experimental process was the same as before. Almost no focal signal was observed in the corresponding region relative to the normal disc of the RGD group.

Compared to normal IVD, the expression of integrin α5β1 was significantly upregulated in the NP of injured IVD at each time point (Figures 2A,B). The radioactivity significantly accumulated in the punctured disc at 1 day postinjury, reached a peak at 1 week postinjury (*p* < 0.01), and gradually decreased from 1 month postinjury (Figures 2A,B). No focal signal was observed in the corresponding disc region of the RGD group (the specific probe for integrin αvβ3) at 7 days postinjury, whereas nonspecific diffusion imaging was observed on the skin and subcutaneous tissue (Figure 2C). These results demonstrated that ^99m^Tc-3PisoDGR2 has high specificity for integrin α5β1.

### *Ex vivo* Confirmation of Integrin α5β1 Expression by IHC

**Figure 3:**
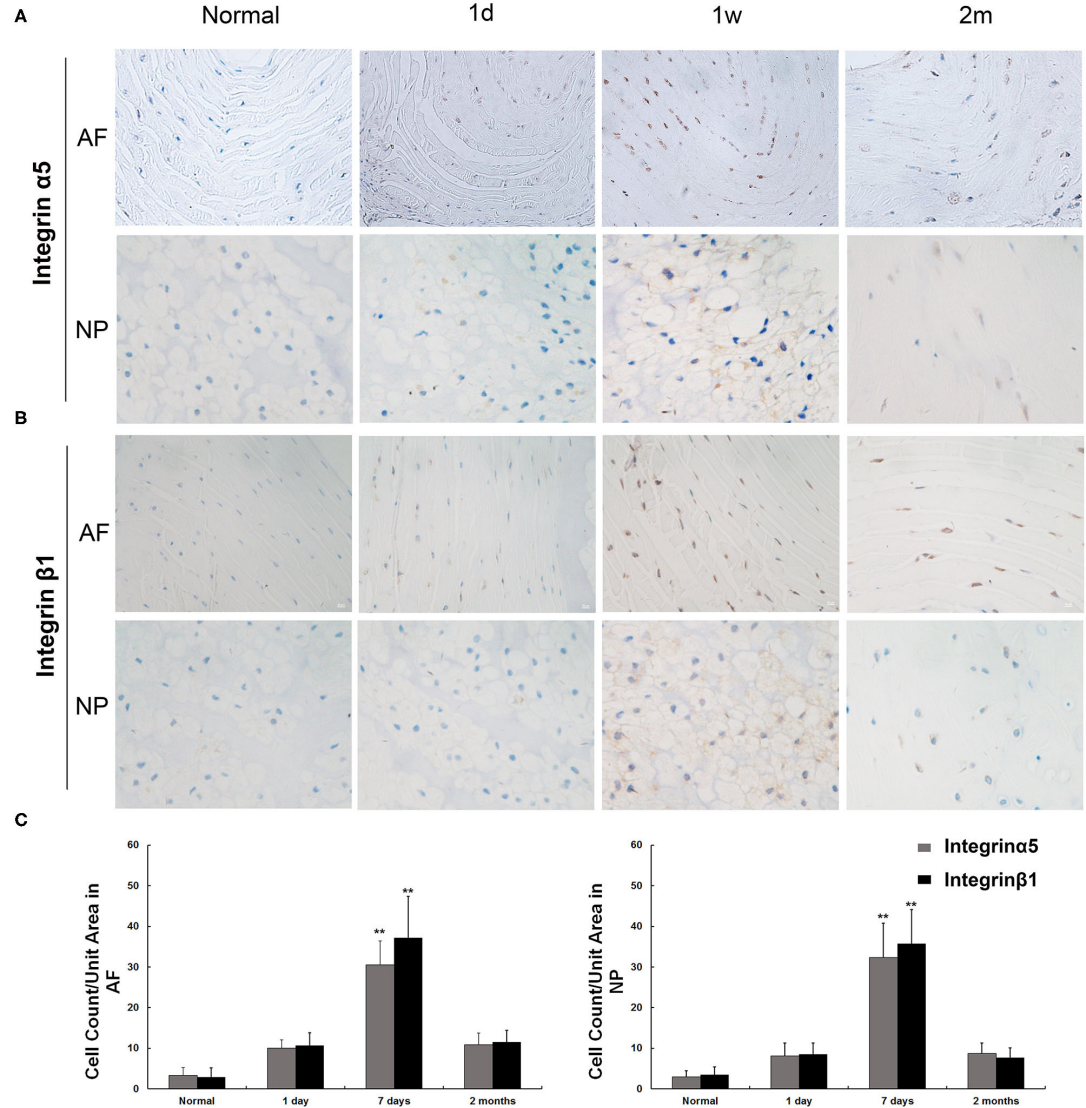
Immunohistochemical staining of integrin α5 and integrin β1 in nucleus pulposus (NP) and annulus fibrosus (AF) cells. **(A)** The expression of integrin α5 was observed in normal AF and NP cells. The increased expression of integrin α5 appeared as early as 1 day postinjury, and the peak appeared at 7 days postinjury and remained visible until 60 days. **(B)** The expression of integrin α5 was observed in normal AF and NP cells. The increased expression of integrin β1 appeared as early as 1 day postinjury, and the peak appeared at 7 days postinjury and remained visible until 60 days. **(C)** Integrin α5- and β1-positive cells per unit tissue volume were plotted at various time points (1 day, 7 days, and 2 months) after rat disc puncture. **p < 0.01 compared to normal disc and injured disc at 1 day and 2 months.

The IHC staining showed that integrin α5 and integrin β1 were expressed on the surface of NP and annulus fibrosus (AF) cells in the control group (Figures 3A,B). In the injured group, the expression levels of integrin α5 and integrin β1 were slightly increased at postoperative day 1. One week after injury, extensive-expression of integrin α5 and β1 was observed on the membranes of NP and AF cells (*p* < 0.01). Two months after the injury, the distribution of NP and AF cells was scattered in the IVD, and the IHC staining on the cell surface was uneven. Moreover, the expression levels of integrin α5 and β1 at 2 months postinjury were decreased but were still higher than those in the control group (Figure 3). The changing pattern of *ex vivo* expression of integrin α5β1 was consistent with that of *in vivo* detection by SPECT/CT.

### *In vivo* Evaluation of Morphological Changes by MRI

**Figure 4:**
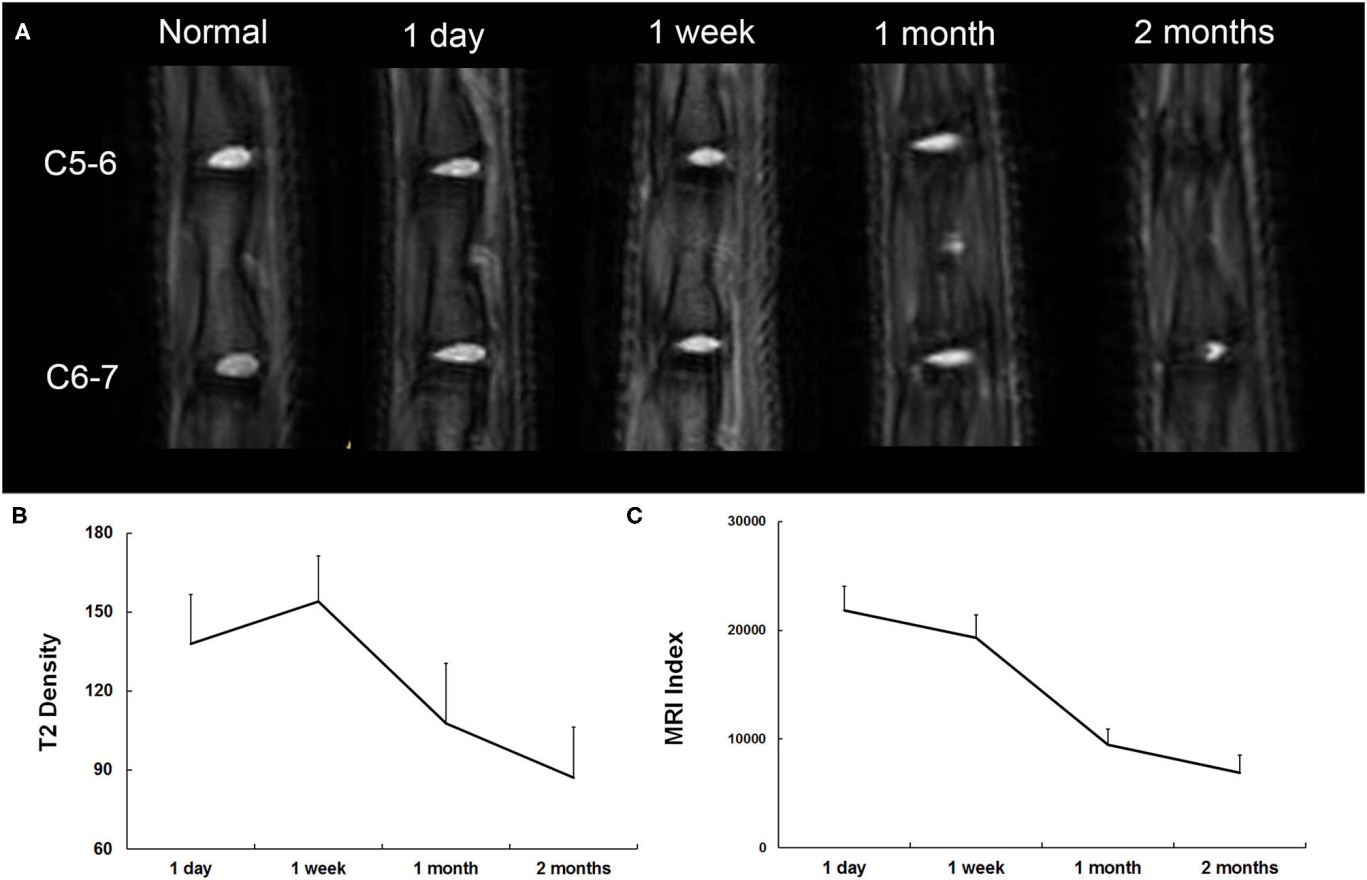
**(A)** Representative serial T2-weighted midsagittal images of the caudal disks 1 day, 7 days, 1 month, and 2 months postinjury. **(B)** Quantitative analysis of T2 density. **(C)** Quantitative analysis of the MRI index.

T2-weighted midsagittal MRI of the caudal disks in a representative case is shown in Figure 4A. At 1 day postinjury, the T2 density was high on the NP and low on the endplate. The adjacent vertebrae were separated, and the height of the intervertebral space was normal. The T2 density continued to increase until 1 week postinjury and then began to decrease after 1 month postinjury. Finally, T2 density reached the lowest with narrowed intervertebral space at 2 months postinjury. The MRI index significantly decreased from 1 week to 2 months postinjury (Figure 4). In contrast to T2 density, the changing pattern of the MRI index was a monotone decreasing curve at each time point (Figure 4C).

### *Ex vivo* Confirmation of Morphological Changes by Histological Staining

**Figure 5:**
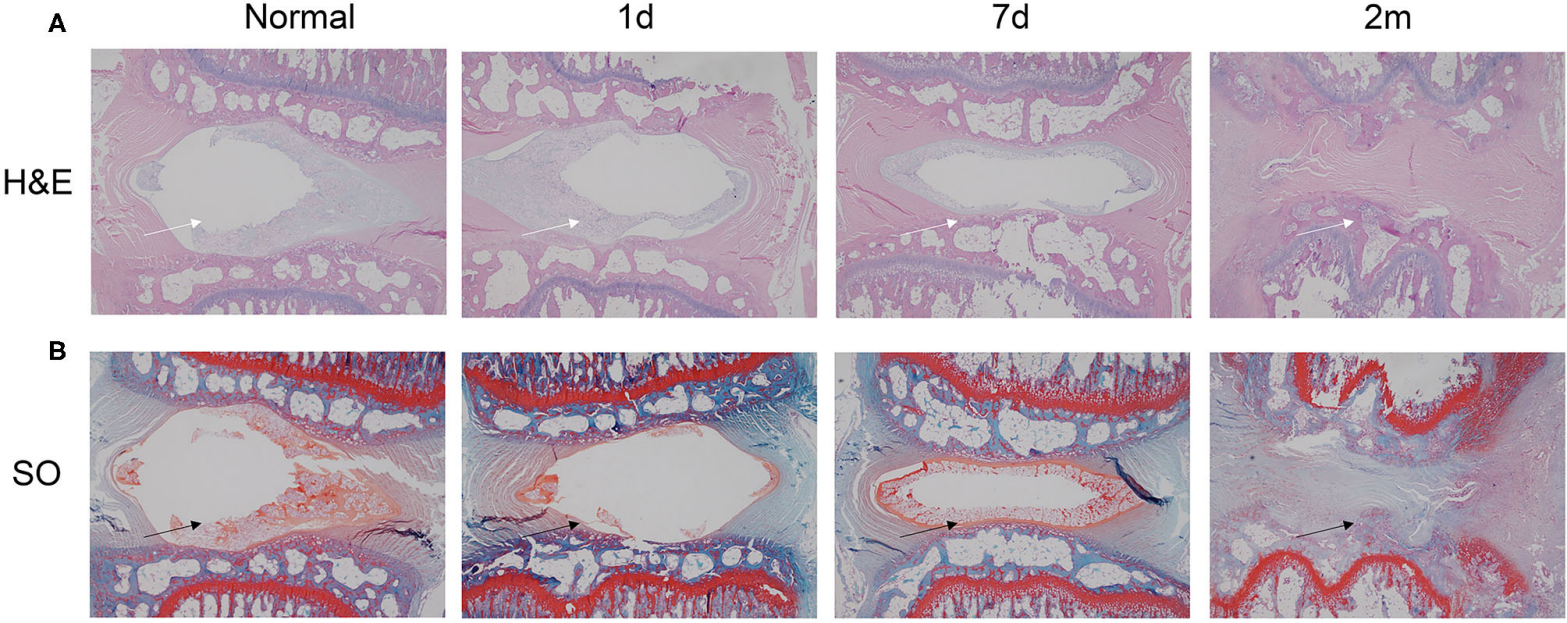
HE and safranin-O staining of injured disks at various time points (1 day, 7 days, 1 month, and 2 months). **(A)** HE staining revealed that the needle puncture induced structural changes (white arrow) in the tissue of the herniated IVD. **(B)** Safranin-O staining demonstrated that after stab perturbation, the positively stained areas became much smaller (black arrow), indicating decreased proteoglycan content. HE, hematoxylin and eosin.

The border between the AF and the NP was determined by HE staining at postoperative day 1 (Figure 5A). However, at 1 week postinjury, the number of notochordal cells and chondrocyte-like cells in the NP began to decrease, and the arrangement of the AF began to become disordered with a blurred border. At 1 month postinjury, the number of notochordal cells and chondrocyte-like cells in the NP was further reduced, and fibroblast-like cells and degenerative necrotic cells emerged. At 2 months postinjury, the NP had almost disappeared and was replaced by fibrocartilage tissue, whereas the arrangement of AF was still disordered with a blurred border. The same changing pattern was also observed with safranin-O staining, indicating decreased proteoglycan (Figure 5B).

## Discussion

Low back pain, a pivotal factor leading to the decline or even loss of the workforce, is a common clinical symptom in modern society that places larger physical, psychological, and economic burdens on patients and their families (22). Owing to the accelerated aging of the population, the number of patients suffering from LBP has continued to increase (23). Disc herniation is acknowledged as one of the most common causes of LBP (24). Occupational epidemiology studies have shown that traumatic injury, heavy physical work, lifting, and excessive straining are important risk factors for disc herniation (25).

The IVD is composed of the upper cartilage endplate, lower cartilage endplate, AF and NP. A normal NP in the IVD has a high matrix content but few blood vessels and nerves. These physiological characteristics suggest that the interplay between IVD cells and the extracellular matrix substantially influences degeneration. Integrins are a class of transmembrane proteins that are widely distributed on the cell surface, connecting cells with the extracellular matrix to maintain the structural and mechanical integrity of the cell. Moreover, integrins transmit mechanical signals and biochemical signals of the extracellular matrix into the cell and are involved in several biological functions, such as cell survival, apoptosis, and gene expression (26, 27). Integrin α5β1 is a classic fibronectin receptor that is mainly expressed in the chondrocytes of adults (28). By linking fibronectin fragments in chondrocytes, integrin α5β1 initiates the downstream cascade signal amplification effect, thereby leading to matrix degradation and remodeling (29). IVD NP cells are similar to articular cartilage cells in terms of tissue origin and composition, and recent studies have shown that such a pathway exists in IVDs (30, 31). Therefore, it is postulated that integrin α5β1 is an important cell membrane receptor for IVD degeneration.

The changes in integrin α5β1 on the surface of IVD cells during disc degeneration remain controversial (32, 33). Kurakawa et al. (32) found that under pressure, the high expression of integrin α5β1 in NP and AF cells is involved in the process of disc degeneration. However, Zhang et al. (34) found that under unbalanced dynamic and static forces, the expression of integrin α5β1 in disc cells decreases, subsequently inducing excessive apoptosis of disc cells. Christine et al. (30) performed IHC on NP cells from normal and degenerated human disks and found that the expression level of integrin α5β1 does not significantly change during disc degeneration. Such inconsistent results may be attributed to different time points and different modeling methods of IVD degeneration. An in-depth understanding of how integrin α5β1 is involved in disc degeneration, thereby leading to excessive apoptosis of disc cells and matrix degradation, will be helpful to shed light on the pathophysiologic progression of disc degeneration.

However, a sensitive, non-invasive, and efficient method has not been developed for the detection of integrin α5β1 in IVD. The traditional detection methods mainly include reverse transcription-polymerase chain reaction (RT-PCR), Western blotting, and IHC staining, which are impracticable for observing real-time changes in expression in the same individual *in vivo*. Current imaging diagnostic techniques to assess the degree of disc degeneration, such as X-rays and MRI, are conducted based on morphological aberrations. Molecular imaging technology can target molecules or biological macromolecules *in vivo* to reflect specific cellular and molecular events (35). Previous studies have used radio-labeled isoDGR dimer peptide to detect integrin α5β1-positive tumors (18), demonstrating good *in vivo* and *in vitro* properties, as well as ideal pharmacokinetic properties, and initial clinical translational research has been conducted (15). Therefore, the present study investigated whether these molecular probes detect degenerative disc diseases, which may provide innovative methods and ideas for the pathogenesis and treatment of disc degenerative diseases, as well as to further expand the application of isoDGR.

In the present study, SPECT imaging of ^99m^Tc-3PisoDGR2 in the IVD injury model demonstrated clear imaging of IVD 30 min after the injection of the imaging agent, indicating that ^99m^Tc-3PRGD2 promptly binds with integrin α5β1 receptors. The distribution of ^99m^Tc-3PisoDGR2 at different time points showed that the expression of integrin α5β1 was induced after the establishment of the acute disc herniation model. With the progression of time, the uptake of imaging agents in the IVD showed an increasing tendency, reaching a peak 1 week postinjury and then gradually decreasing. Nevertheless, the imaging agent content remained at a high level until 2 months postinjury. The expression of integrin α5β1 receptor in IVD was analyzed using IHC, and the results agreed with the uptake trend, which may be attributed to the molecular mechanism during the degeneration of IVD.

The high expression of integrin α5β1 during the acute stage after disc injury plays a key role in initiating the downstream signal cascade amplification effect. After the integrin molecules bind to fibronectin fragments in the extracellular matrix, the allosteric activation of integrin is initiated to regulate focal adhesion maturation, remodel the actin cytoskeleton, and mediate “outside-in” signal transduction. Moreover, fibronectin fragment signals serve to activate the intracellular components and initiate “inside-out” signal transduction, thereby inducing upregulation of integrin in the vicinity of the focal adhesions, facilitating more effective sensing of changes in the cell-matrix and coordinating signal transduction. Such an underlying mechanism provides the possibility for clinical practice, i.e., the expression of integrin α5β1 receptor can be quantitatively analyzed by non-invasive examination to help with the evaluation of the stage of disc degeneration and the effect of clinical intervention.

The present study had several limitations. First, degeneration of the IVD is caused by a variety of complex factors, such as heredity, traumatic injury, and overloading stress. Second, the animal model established in this experiment only simulated acute disc herniation with AF damage after traumatic injury. Although the model has many similarities with human disc herniation regarding clinical manifestations, pathology, and immunology, it does not precisely simulate the chronic progression of IVDs in the human body. Third, patients with severe degeneration of IVDs are commonly observed in clinical practice; their IVDs have been severely degraded, and their NP may have atrophied and disappeared. It is unknown whether the same conclusion can be made regarding these patients. Thus, further studies are needed for more precise validation. Finally, integrin α5β1 is not a cost-effective method for asymptomatic or slightly symptomatic patients who can easily be followed-up with MRI, which is less expensive and easier to perform.

## Conclusions

In the present study, a novel radioactive molecular probe, called ^99m^Tc-3PRGD2, was used for the first time in an animal model of acute disc herniation to preliminarily evaluate its diagnostic value in acute disc herniation. Through real-time observation *in vivo*, the use of ^99m^Tc-3PRGD2 well reflected the expression pattern of integrin α5β1 in acute disc herniation. As acute disc herniation progressed, the uptake of ^99m^Tc-3PRGD2 reached a peak at 1 week postinjury. Our data established a correlation between the uptake of ^99m^Tc-3PRGD2 in acute disc herniation and the expression of integrin α5β1, providing a solid theoretical basis for understanding the pathophysiology of acute disc herniation, developing a novel drug treatment, and providing a more precise clinical monitoring of the curative effects.

## Data Availability Statement

Reasonable requests to the available datasets should be directed to the corresponding author(s).

## Ethics Statement

The animal study was reviewed and approved by Xuanwu Hospital. Written informed consent was obtained from the owners for the participation of their animals in this study.

## Author Contributions

JG: writing—original and draft data curation. CY: writing—review and editing. XT: animal support. LC: software. HG: methodology. QY: software and draft data curation. XW: language editing. HW and ZC: resources. FJ: writing—review and editing and project administration. All authors contributed to the article and approved the submitted version.

## Funding

This study was supported by Beijing Municipal Science and Technology Commission (Grant number: Z191199996619048) and Beijing Municipal Commission of Education (Grant numbers: KZ202010025043 and 1192070315).

## Conflict of Interest

The authors declare that the research was conducted in the absence of any commercial or financial relationships that could be construed as a potential conflict of interest.

## Publisher's Note

All claims expressed in this article are solely those of the authors and do not necessarily represent those of their affiliated organizations, or those of the publisher, the editors and the reviewers. Any product that may be evaluated in this article, or claim that may be made by its manufacturer, is not guaranteed or endorsed by the publisher.
